# Supplementary figures and images for: Modulation of SUR1 KATP Channel Subunit Activity in the Peripheral Nervous System Reduces Mechanical Hyperalgesia after Nerve Injury in Mice
Source: Int J Mol Sci. 2019 May 7;20(9):2251. doi: 10.3390/ijms20092251 (PMC6539735; doi:10.3390/ijms20092251)

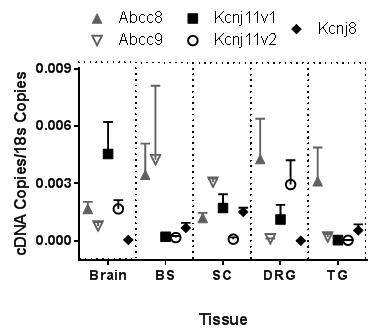

Supplement: Supplementary file 1 [file ijms-20-02251-s001.zip › Supplemental Figures 1-3/KATP CNS PNS SNL Supplemental Figure 1 March 2019.tif]

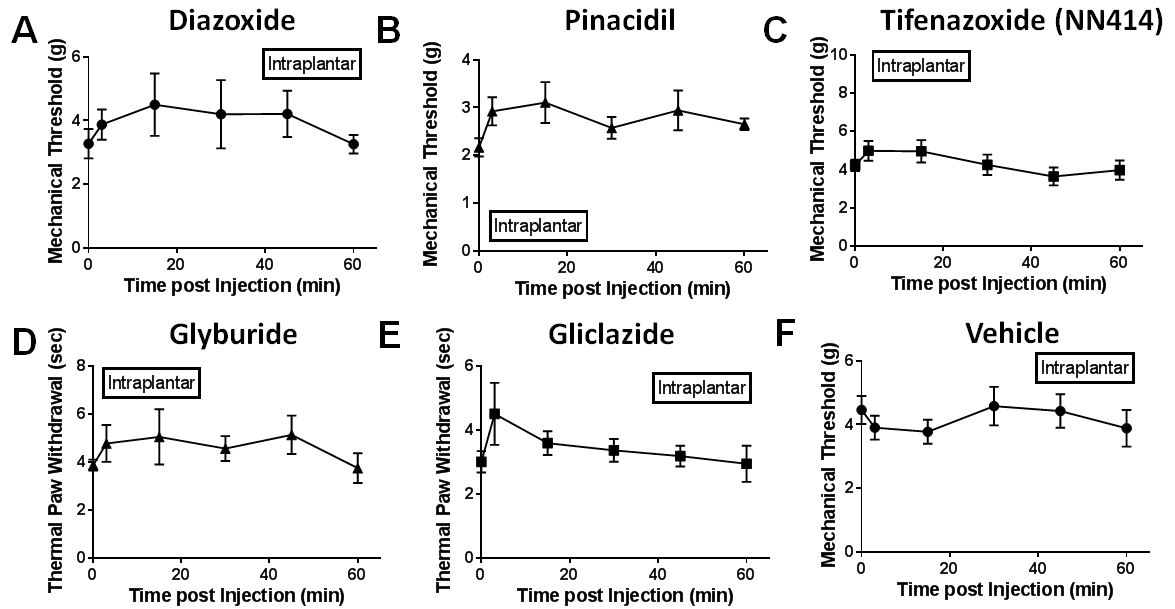

Supplement: Supplementary file 1 [file ijms-20-02251-s001.zip › Supplemental Figures 1-3/KATP CNS PNS SNL Supplemental Figure 2 March 2019.tif]

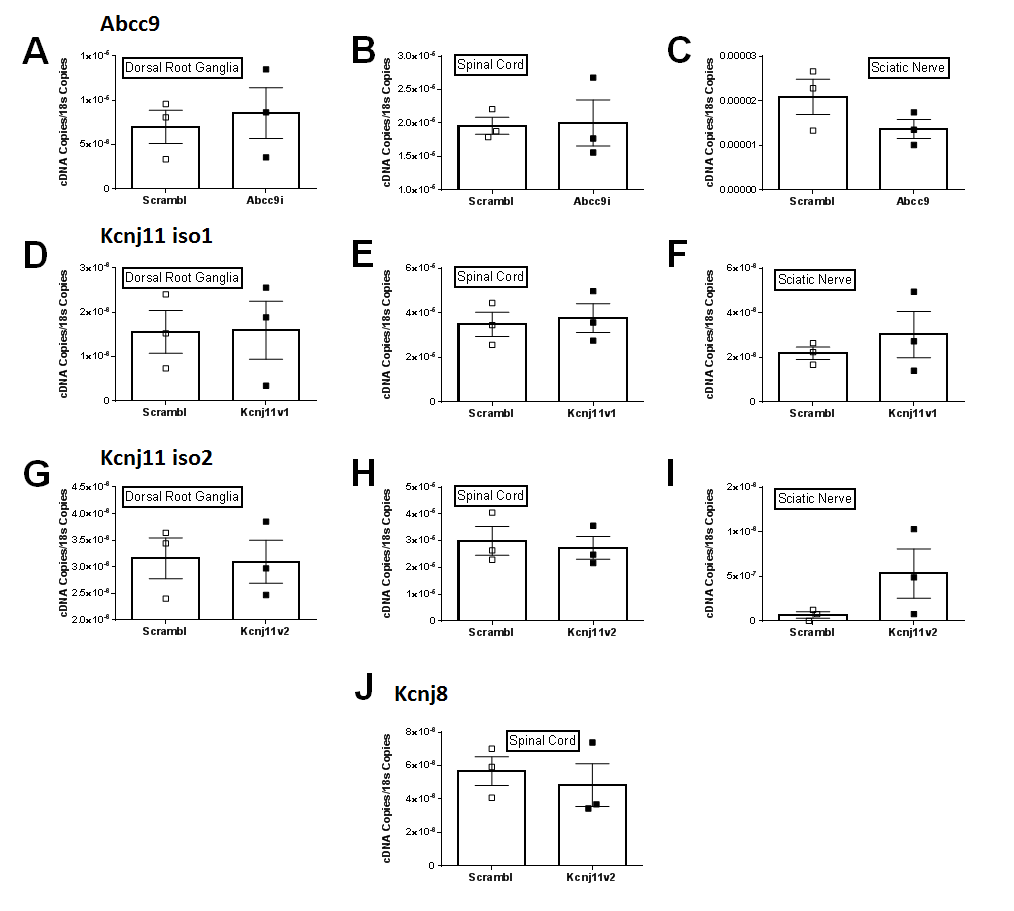

Supplement: Supplementary file 1 [file ijms-20-02251-s001.zip › Supplemental Figures 1-3/KATP CNS PNS SNL Supplemental Figure 3 March 2019.tif]
